# Experience shapes non-linearities between team behavioral interdependence, team collaboration, and performance in massively multiplayer online games

**DOI:** 10.1038/s41598-024-57919-w

**Published:** 2024-04-03

**Authors:** Carlos Carrasco-Farré, Nancy Hakobjanyan

**Affiliations:** 1 Toulouse Business School, Toulouse, France; 2 Amazon Web Services, Hamburg, Germany

**Keywords:** Human behaviour, Computational science

## Abstract

This paper examines quantitative predictors of team performance in Massively Multiplayer Online Games (MMOGs) based on team management literature. Analyzing data from more than 140,000 squad-mode matches involving over 500,000 players, we replicate and extend existing research by confirming a curvilinear association between behavioral interdependence and team performance and introduce the moderating effect of experience. For less experienced teams, behavioral interdependence follows an inverted U-shaped pattern showing that excessive collaboration may be counterproductive. However, this is not the case for experienced teams, where the relationship is fairly linear. Additionally, we observe that riskier teams tend to perform worse. Moreover, our research also highlights the potential of e-sports data in advancing behavioral science and management research. The digital nature of e-sports datasets, characterized by size and granularity, mitigates concerns related to reproducibility, replicability, and generalizability in social science research, offering a cost-effective platform for scholars with diverse backgrounds.

## Introduction

Social sciences are suffering a “replication crisis”^1^. Disciplines like psychology, team research, organizational behavior, and behavioral economics have faced mounting concerns related to the reproducibility^2^, replicability^3^, generalizability^4^, and overall trustworthiness of their findings^5^ usually because of external validity issues in experimental research and limited sample sizes in observational studies^1^. Therefore, we need complementary data sources that can help researchers not only replicate previous results but also to expand them^6^. In this paper we argue that video games, as interactive digital experiences, have transcended mere entertainment to serve as a unique platform for researchers to delve into intricate domains of human behavior^7^.

In the United States alone, more than 215 million people engage in video games for at least one hour per week, with approximately 75% of all households having at least one active player^8^. Interestingly, a significant proportion of video game players, approximately 83%, actively participate in multiplayer modes that heavily rely on teamwork and collaboration^8^. Therefore, the widespread availability and immense popularity of video games has triggered an exceptional opportunity to study decision-making processes regarding collaborative dynamics.

In the management literature, the concept of behavioral interdependence refers to the extent to which team members actively cooperate in task-solving^9^. Essentially, behavioral interdependence gauges the level of interaction and interconnection among team members^10^, with a significant indicator being the voluntary provision of assistance between teammates during task completion^11^. Despite the construct is positively associated with team performance, there is also evidence that the relationship between behavioral interdependence and team performance is curvilinear^12^, aligning with the “*too much of a good thing effect*” described by Pierce and Aguinis^13^.

Pierce and Aguinis argued that scholars often assume linear relationships for the sake of simplicity, but in reality, beneficial factors can reach inflection points where their impact on desired outcomes becomes nonlinear and potentially adverse^13^. Exceeding these inflection points may result in suboptimal performance. In other words, over-emphasizing collaboration can lead to team members sacrificing their individual goals to prioritize others, which may not always contribute positively to overall team performance^14^. These curvilinear relationships have been observed in team cohesion^15^, team communication^16^, team size^17^ or team conflict^18^ among others. In summary, previous literature emphasizes the importance of team members striking a balance between competing priorities, seeking an equilibrium between behavioral autonomy and behavioral interdependence among teammates^19,20^. However, there have been recent calls to explore moderators that may affect curvilinear relationships towards team performance^15^.

In videogames, one of the main drivers of players performance is their experience^7^. However, it is little known to what extent players experience may moderate the curvilinear relationship between team behavioral interdependence and team performance^15^. Therefore, the purpose of this study is threefold. First, to confirm the curvilinear relationship between team behavioral interdependence and team performance in a different environment (therefore increasing the generatability of previous findings through replication). Second, to extend previous findings by examining the moderating role of experience in this curvilinear relationship^15^. Third, to prove the potential of video games as empirical contexts that can provide big -and free- sample sizes that are difficult to achieve through traditional data collection methods like observational studies or laboratory experiments^6^. By doing so, we contribute to recent calls to mitigate the replication crisis in management research^1^, we expand the existing results^15^, and we provide evidence that video game data can be leveraged for behavioral studies in social science disciplines.

Out results indicate strong support for the positive association between behavioral interdependence and team performance, with a curvilinear relationship observed. High behavioral interdependence predicts positive team performance, but maintaining a loose collaboration within high interdependence levels also offers benefits depending on experience. Experience moderates this relationship: excessive collaboration can be counterproductive for low-experience teams, while the relationship becomes more linear for high-experience teams.

## Empirical context

Within the realm of team-based competitive Massively Multiplayer Online Games (MMOGs), players are presented with a multitude of individual and collective decisions that significantly impact their overall performance. Amidst honing their individual skills, players face a pivotal choice that shape their performance—the decision to collaborate with others team members while navigating through contextual factors such as time-pressure, resource constraints, and interactions with other teams.

PUBG is one of these MMOGs in the battle royale shooter genre. The game has garnered an astonishing player base of over 400 million worldwide, solidifying its position as the 5th best-selling game of all time^21^. Its mechanics are the following: 100 players are grouped into teams of 4 and dropped onto an island, with the ultimate objective of outlasting other teams through resource gathering (weapons, armors, and boosters) and intense player-versus-player combat. Players have the option to choose from a selection of maps, each with varying sizes, and decide with whom they want to play or let the videogame assign them to a random team. The available maps include Miramar and Erangel, both measuring 8 × 8 km; Sanhok, a 4 × 4 km map; and Karakin, a 2 × 2 km map. In this virtual battleground, distance is measured in centimeters, and coordinates can span from 0 to 816,000 cm, 0–408,000 cm, or 0–204,000 cm on both the longitude and latitude axes. As the match begins, players strategize their landing spots while aboard the same plane, setting the stage for a competitive gameplay experience^22^.

Once all players have landed in their desired spots, and as the match unfolds, the playing area gradually shrinks, compelling teams to engage with each other. In the beginning, players have a 5-min window to explore the vast map and scavenge for resources. After this initial phase, the playing area starts to contract at a constant rate of 22.8 m per second. When the area reaches a specific diameter, players are granted an additional 3 min and 20 s to explore before the shrinking process resumes. This cyclic pattern persists until the match reaches a total duration of 30 min. The controlled nature of the game, with fixed player counts and identical starting conditions, ensures that beyond individual skills (experience), successful teamwork and strategic risk-taking play pivotal roles in achieving victory^23^. This provides an excellent opportunity to explore the interplay of behavioral interdependence, experience, and team performance under high-stress, competitive circumstances.

## Results

### Model performance

**Figure 1:**
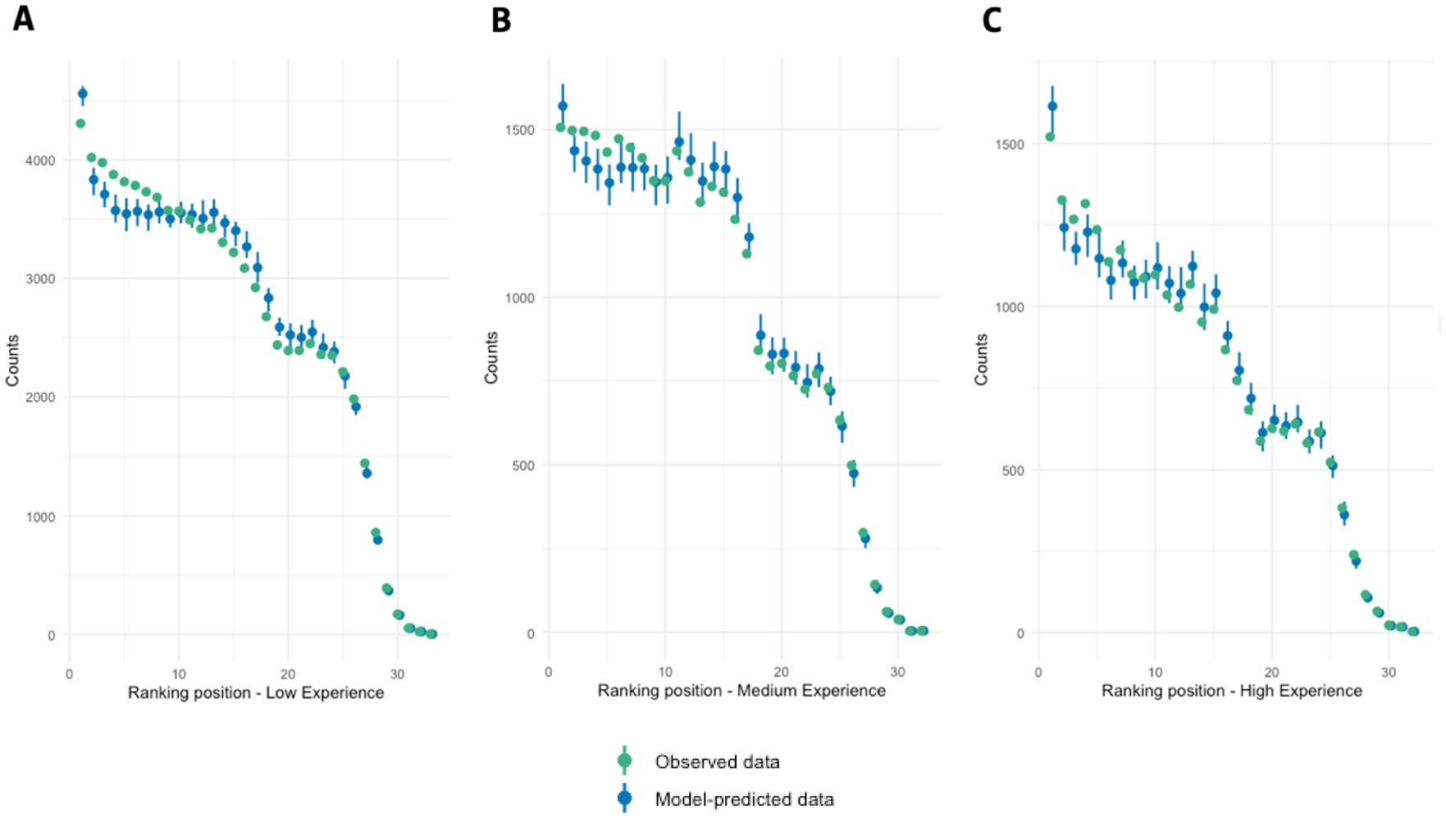
Predicted VS Observed data in ordinal models; (**A**) ordinal model A (low experience), (**B**) ordinal model B (medium experience), (**B**) ordinal model C (high experience).

Figure 1 depicts the predictive power of the different models used in our study, with all of them providing similar performance. The r2ML for the low experience sample is 0.356, for the medium experience sample is 0.349, and for the high experience sample is 0.352. These results indicate that the models are reasonably effective at explaining the variability in ranking positions (see Fig. 1 for a graphical representation). For the understanding of the ordinal models, it is worth noting that the coefficients presented in the Table 1 should be interpreted in the light of an ordinal dependent variable based on ranking positions. Therefore, a negative coefficient estimate is associated with a higher position in the ranking, while a positive one is associated with a lower position in the ranking.

**Table 1:** Ordinal regression results.

	Full Sample 1	Full Sample 2	Low experience	Medium experience	High experience
High Behav. Interdependence	− 12.112***	− 20.838***	− 11.427***	− 12.760***	− 13.655***
	(− 12.385, − 11.839)	(− 21.095, − 20.580)	(− 11.786, − 11.068)	(− 13.327, − 12.192)	(− 14.298, − 13.013)
Medium Behav. Interdependence	− 2.541***	− 12.473***	− 0.275	− 5.585***	− 5.817***
	(− 2.887, − 2.195)	(− 12.812, − 12.134)	(− 0.727, 0.177)	(− 6.303, − 4.867)	(− 6.642, − 4.993)
Low Behav. Interdependence	2.868***	− 13.702***	4.842***	− 0.665	− 0.774
	(2.454, 3.282)	(− 14.113, − 13.292)	(4.312, 5.372)	(− 1.549, 0.219)	(− 1.800, 0.251)
Low collaboration	− 2.314***	− 2.310***	− 3.153***	− 1.322***	− 1.035***
	(− 2.409, − 2.219)	(− 2.405, − 2.216)	(− 3.279, − 3.026)	(− 1.512, − 1.131)	(− 1.248, − 0.822)
High collaboration	− 1.980***	− 1.990***	− 2.377***	− 1.192***	− 1.503***
	(− 2.095, − 1.865)	(− 2.105, − 1.875)	(− 2.528, − 2.226)	(− 1.429, − 0.955)	(− 1.774, − 1.232)
High landing risk	− 0.005***	− 0.005***	− 0.005***	− 0.004***	− 0.004***
	(− 0.005, − 0.004)	(− 0.005, − 0.004)	(− 0.005, − 0.005)	(− 0.005, − 0.003)	(− 0.005, − 0.003)
Low landing risk	− 0.005***	− 0.007***	− 0.005***	− 0.002	− 0.005***
	(− 0.006, − 0.003)	(− 0.009, − 0.006)	(− 0.007, − 0.003)	(− 0.005, 0.001)	(− 0.007, − 0.002)
High risk	0.033***	0.032***	0.035***	0.030***	0.030***
	(0.032, 0.034)	(0.031, 0.033)	(0.033, 0.036)	(0.028, 0.032)	(0.027, 0.032)
Low risk	0.002*	− 0.001	0.002	0.004	0.004
	(− 0.000, 0.005)	(− 0.003, 0.001)	(− 0.001, 0.005)	(− 0.001, 0.008)	(− 0.001, 0.010)
High Behav. Interdependence—Quadratic		45.598***			
		(45.525, 45.671)			
Medium Behav. Interdependence—Quadratic		103.198***			
		(103.155, 103.240)			
Low Behav. Interdependence—Quadratic		162.365***			
		(162.325, 162.404)			
Observations	140,699	140,699	85,388	30,645	24,666

Note: *p < 0.1; **p < 0.05; ***p < 0.01.

The results of the ordinal regression models align with previous evidence stating that “High Behavioral Interdependence” is positively associated with team performance (in absolute values to account for ranking order: *β* = 12 ranking positions overall; *p* < 0.01) while “Low Behavioral Interdependence” is negatively associated with team performance (*β* = − 2 ranking positions overall; *p* < 0.01). Furthermore, both “High Collaboration” and “Low Collaboration” variables have negative coefficient estimates, indicating that an increase in the maximum or minimum distance between players, is associated with an increase in the team’s overall position in the ranking. However, the magnitude of the effect is greater for “Low Collaboration” (*β* = − 2.31 overall; *p* < 0.01) compared to “High Collaboration” (*β* = − 1.98; *p* < 0.01). Overall, these results suggest that maintaining an optimal level of behavioral interdependence is crucial for team performance. While keeping a high behavioral interdependence can positively contribute to team performance, having a loose high behavioral interdependence contribute even more positively. This finding is confirmed by the quadratic terms and their effect direction, where we observe a positive and statistically significant (*p* < 0.01) result for all levels of behavioral interdependence. Besides identifying a curvilinear effect, our results also indicate that “Low Behavioral Interdependence” has the largest magnitude for the quadratic term (*β* = − 162.36; *p* < 0.01), and “High Behavioral Interdependence the smallest quadratic effect (*β* = − 45.60; *p* < 0.01). This suggests that as behavioral interdependence decreases (moving from high to low), the curvilinear effect becomes more “stepped” or pronounced. Overall, these results mean that, when trying to maximize performance, teams should collaborate closely, but up to a point.

**Figure 2:**
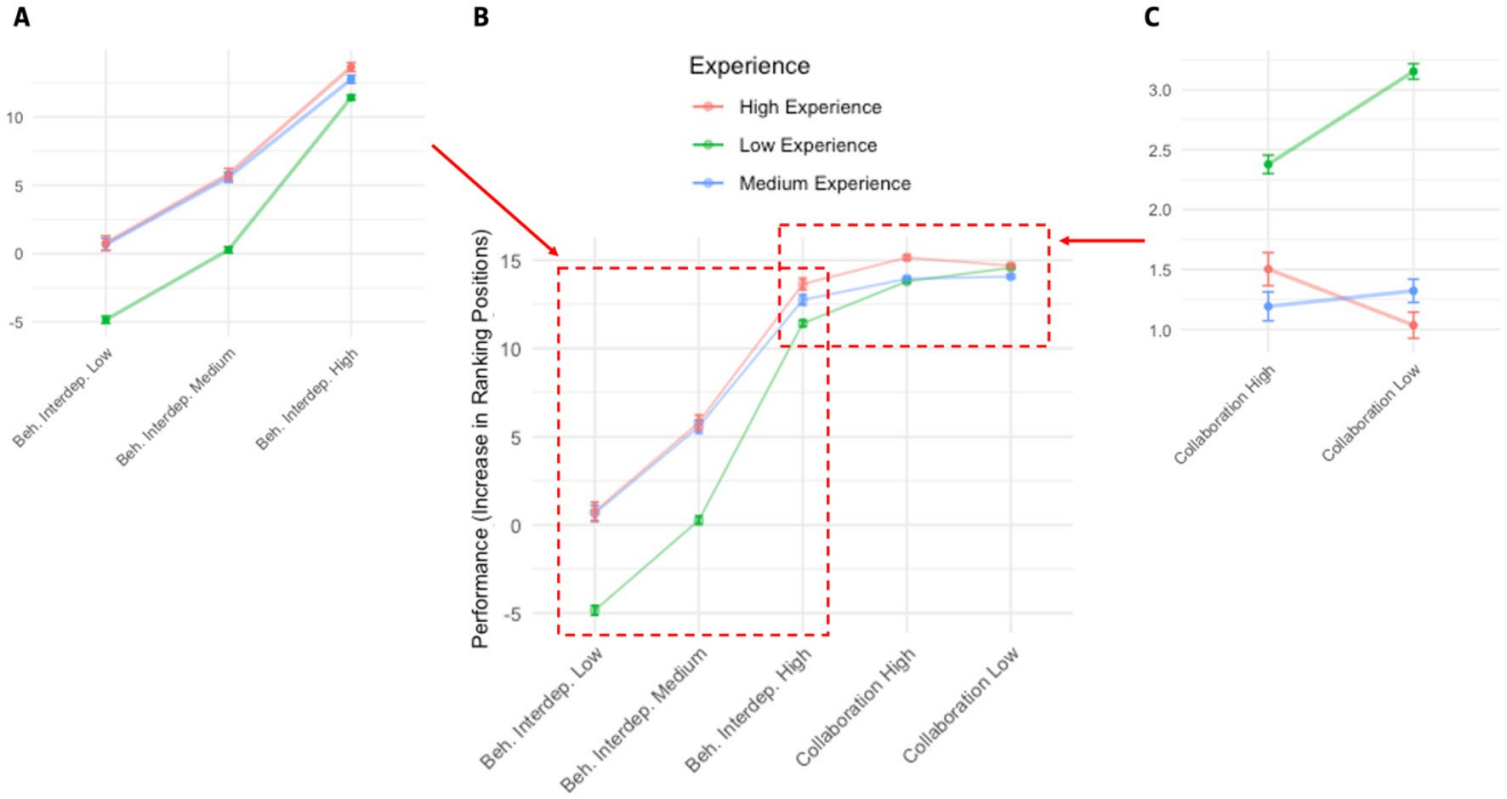
(**A**) Effect of behavioral interdependence at different levels of experience. The higher the behavioral interdependence of a team, the higher their ranking position. (**B**) The join effect of behavioral interdependence and team collaboration. (**C**) Effect of high and low collaboration (tight vs loose behavioral-interdependence) at different levels of experience.

The results of behavioral interdependence at different levels of experience (Fig. 2A) replicate those observed at the aggregate level. As players increase behavioral interdependence, their performance also increases. However, this is contingent to the experience level of the players. For low experienced players, having a low behavioral interdependence has a negative effect (with an average decrease of 4.8 ranking positions [± 0.27]). In contrast, more experienced players do not suffer from such a penalty (medium experience average gain of 0.66 positions [± 0.45] and high experience average of 0.77 ranking positions [± 0.52]). In other words, the difference between low and high behavioral interdependence in low experience teams is higher (16 positions) than the difference for highly experienced teams (13 positions).

As for the curvilinear relationship, the stratified effect of experience also yields contrasting results (Fig. 2C). While loose behavioral interdependence (low collaboration) is positively associated with team performance in the case of low experienced teams (tight interdependence = 2.37 ranking positions [2.29, 2.45] vs loose interdependence 3.15 positions [3.09, 3.21]), it is not significant for medium experienced teams (tight interdependence = 1.19 ranking positions [1.07, 1.31] vs loose interdependence 1.32 positions [1.22, 1.42], and negatively associated with team performance in the case of highly experienced teams (tight interdependence = 1.50 positions [1.36, 1.64] vs loose interdependence = 1.03 ranking positions [0.92, 1.14]). In other words, while a statistically significant inverted U-shape is observed in the case of low experience teams, a U-shaped curvilinear relationship is observed in experienced teams (also statistically significant). However, we do not observe and statistically significant curvilinear relationship in the case of medium experienced teams. These two patterns lead to the results depicted in Fig. 2B. In there, we can observe that a low experienced team with high behavioral interdependence and low collaboration has the same performance (14 ranking positions [13.9, 14.1]) as an experienced team with high behavioral interdependence and low collaboration (14 ranking positions [13.89, 14.11].

As for risk, landing in a high-risk area is positively associated with performance in all ordinal models (*β* = − 0.005; *p* < 0.01 for the full sample models and *β* = − 0.004; *p* < 0.01 for the stratified models). However, we observe that landing in a low-risk area is also positively associated with performance (*β* = − 0.005; *p* < 0.01 and *β* = -0.005; *p* < 0.01 for the full sample models and *β* = − 0.005, *p* < 0.01 for the low experience and high experience stratums, for the medium experience stratum the results are not significant).

However, the previous results also suggest that the effect sizes of the risk variables are much smaller compared to those of the behavioral interdependence. While risk variables show a positive association with team performance, their coefficient estimates are considerably smaller in magnitude compared to the behavioral interdependence variables. These findings highlight the relative importance of behavioral interdependence as a factor influencing team performance compared to the risk variables. All these results are corroborated in our robustness checks using logistic models (see Annex for detailed results).

## Discussion

In our paper, we present an analysis of quantitative behavioral predictors of team performance, drawing insights from team management literature. Our findings strongly support the positive association between behavioral interdependence and team performance in the context of MMOGs. While high behavioral interdependence is a significant predictor of positive team performance, our models reveal that maintaining a loose interdependence within behavioral interdependence levels also offers performance benefits depending on the experience level. Our results indicate that experience moderates the curvilinear relationship between behavioral interdependence and team performance, showing that excessive behavioral interdependence can be counterproductive at low levels of experience. However, when experience is high, the curvilinear relationship flattened and became more linearly associated with team performance. In essence, the optimal strategy for maximizing team performance involves striking a balance between loose and tight collaboration within high behavioral interdependence depending on the experience level. This result sets the scene for our first contribution: a replication of the curvilinear relationship between behavioral interdependence and team performance.

This is not a superficial contribution, since the evidence presented in this paper not only confirms, but also extends the existing one, two of the objectives of replication studies^6^. More specifically, replicating previous results is important because management research is under a “replication crisis”^1^, which means that our paper contributes to the recently growing efforts of replication of previous results^24,25^. Moreover, we do so through methodological triangulation^26^, which consists in proving evidence regarding the consistency of previous results through different methods and data^27^.

Second, our contribution regarding replication is not only by proving consistent results, but we also made a contribution by extending previous findings through the moderating effect of experience. Our results demonstrate that experience plays a key role in determining the curvilinear effect of behavioral interdependency and team performance, providing evidence for an inverted U-shape in the case of low experienced teams, and evidence of a linear relationship in the case of high experienced teams (and inconclusive results for the medium experienced teams). These findings can be explained by several theories.

The Cognitive Load Theory suggests that individuals have a limited capacity for processing information. For low-experienced teams, a loose level of high behavioral interdependence may reduce cognitive load and the *too much of a good thing effect*^13^ by providing structure without overwhelming them. This strategy may improve their learning process and facilitate their adaptation to their tasks within the team, granting them more autonomy and flexibility in their roles, which can reduce the cognitive load of low experience players^16^, and therefore increase their chances of attaining higher ranking positions. Conversely, high-experienced teams, with their greater capacity to handle complex information, may benefit more from tight levels of high behavioral interdependence as they can process and coordinate more effectively^28^. Similarly, the Social Facilitation Theory posits that the presence of others can enhance performance on simple tasks but hinder performance on complex tasks^29^. In the context of experienced teams, the tight levels of high behavioral interdependence between team members may enhance performance as tasks are more within their realm of expertise. In contrast, for less experienced teams, tight collaboration and high interdependence might initially overwhelm and hinder performance until they gain more experience and the tasks become 'simpler' for them^30^.

On the other hand, our results could also be explained by the Self-Determination Theory, which emphasizes the role of autonomy in fostering motivation and performance^31^. For less experienced teams, too tight collaboration might impinge on their sense of autonomy, leading to lower motivation and performance. As they gain experience, they might become more comfortable with tight collaboration, seeing it as supportive rather than controlling, thus enhancing their motivation and performance^31^. This potential explanation also aligns with the Organizational Learning Theory^32^. Initially, low-experienced teams may struggle with tight collaboration and high interdependence due to a lack of common knowledge acquired through experience. However, as they gain experience, their ability to effectively collaborate and benefit from tight collaboration and high interdependence increases. While the specific mechanisms behind the observed U-shaped relationships in team behavior and performance remain unidentified, theories such as Cognitive Load Theory, Social Facilitation Theory, Self-Determination Theory, or Organizational Learning Theory offer promising avenues for future research. Investigating these aspects could unveil deeper insights into the nuanced relationship between team experience and behavioral interdependence in MMOGs, providing valuable contributions to both theoretical understanding and practical application in the e-sports context.

Moreover, our research establishes the bidirectional fertility between behavioral studies and e-sports as a contextual setting for quantitative studies, offering researchers an opportunity to address some of the limitations in social science research. Over the past fifteen years, social science disciplines like psychology, team research, organizational behavior, and behavioral economics have faced mounting concerns related to the reproducibility^2^, replicability^3^, generalizability^4^, and overall trustworthiness of their findings^5^. The digital nature of e-sports provides access to huge databases that encompass the decisions made by millions of players helping to mitigate, at least, concerns regarding sample sizes. However, these databases are not just beneficial for research because of their size, but also because of their quality. Everything happening in videogames is stored with high granularity, often a condition not found in physical sports data^33^, which allows for exploration of core theoretical constructs in social sciences.

Furthermore, with the pool of unexplored significant effects diminishing, researchers are redirecting their focus towards investigating smaller effects and the boundary conditions of established effects^34^. While this situation is natural in any scientific discipline after decades of research, this shift requires the utilization of larger sample sizes to ensure that studies have the necessary statistical power to detect smaller effects. However, this increase in sample sizes, representative samples, field studies, etc. to secure statistical power have dramatically increase the cost of publishing behavioral research. Again, the availability, size, and granularity of e-sports data can help mitigating this challenge.

An increasing concern revolves around the potential detrimental consequences of the escalating costs, which could exacerbate the advantages enjoyed by scholars affiliated with well-endowed institutions, while concurrently restricting the prospects for marginalized scholars to make meaningful contributions. This is problematic because a discipline flourishes when it provides a platform for scholars with diverse backgrounds to express their viewpoints and contribute to our collective understanding human behavior^35^. Our paper shows that e-sports can serve to analyze highly complex contexts while maintaining experimental control^36^, ecological validity^37^, and reproducibility^38^, offering great potential for advancing behavioral research at a fraction of the cost compared to the same sample sizes in observational or laboratory studies.

Furthermore, studying e-sports data allows us to examine team dynamics as a bottom-up process emerging from individual behaviors and their interactions^39^, aligning with the perspective of teams as complex adaptive systems^40,41^. This approach provides valuable insights into team behavior and performance in a dynamic and ever-changing environment, offering significant contributions to the understanding of team processes. Indeed, there is a whole stream of research conducted using physical sports data. For instance, Lehman and Hahn^42^ utilize NFL games to study how momentum shapes organizational risk-taking, and Sobrepere i Profitós et al.,^43^ analyze over 2,300 NFL games to understand how individuals regulate risk-taking based on performance feedback. Despite these efforts, we believe that e-sports can provide even more understanding of human behavior. With millions of people worldwide facing various challenges set by game developers and employing diverse strategies to overcome them, the data collected from these interactions presents an invaluable opportunity for scholars to gain a deeper understanding of topics that have been lacking sufficient data for investigation.

The controlled nature of e-sports makes it an ideal environment to empirically test predictions across a wide range of management topics^44,45^. Leveraging this new and evolving phenomenon can also lead to the development of novel theories as researchers explore new contexts for their studies^9,46^. This is the case when e-sports are used, not only to replicate previous results, but also offer the opportunity to provide new empirical and theoretical contributions.

## Limitations and future research

Despite the quantitative evidence backing up our results, we should also take into account several limitations of our analysis. First, the case of generalizability. A potential lack of external validity may be a concern due to the nature of our study context and how the results generalize to other contexts. To mitigate this issue, we propose boundary conditions to state where we think the results may also apply besides behavior of PUBG players^47^. We do so by relying on the similarities with papers using sports data^33^, stating that our findings could be generalizable to highly-competitive team tasks like sales, accounting, or consulting. Moreover, our findings are more generalizable in situations where experience plays a key role and where task require highly interdependent teams, like in the case of professional services that rely on high performance teams^48^. In other words, our results are informative and relevant for organizations that rely on high performance teams, tackling with tasks that require high interdependence, and that perform tasks that are easier to perform as team members gain experience (that is, a low volatile environment). Secondly, due to privacy restrictions in the API, we are not able to control whether the players were communicating through audio. While this could pose a threat to our analysis, based on the robustness checks (see “Methods” section) we believe that this is not an issue in our sample. The reason is that just 0.5% of the players have played together, and therefore we assume it is unlikely that they communicated through audio with other players. Moreover, the countries with more PUBG players are India, US, Russia, Japan, South Korea, China, Finland, Thailand, Canada, and Norway, which means that it is highly unlikely that they speak the same language^21^. Moreover, we believe that the absence of audio data doesn't diminish our ability to deduce coordination and strategy in a gaming context. Instead, we can infer these elements from players' movements, which act as a tangible representation of any verbal strategy, if one exists. This compliance or lack thereof not only informs us about individual player behavior but also sheds light on the team's collective capacity to work cohesively under strategic directives, whether those directives are communicated through audio or rely on other information available to each player, such as teammate locations and more. While we may not directly observe audio communication and potential negotiations regarding the team strategy, we can still gauge the outcomes by tracking the geolocation of players. This information allows us to assess whether these behaviors are effectively coordinated, whether verbally or through other means of information exchange.

Finally, despite the previous limitations, we do believe that e-sports data has a lot of potential for management research. To elaborate on it, we have outlined a list of future research topics that can be effectively analyzed using the same or similar data. This offers exciting possibilities for advancing knowledge and understanding in management research.

### Team cohesion

Team cohesion, as defined by Shaw^49^, refers to the degree of motivation among team members to remain a part of the team. High cohesion within a team has numerous benefits, including enhanced member coordination during team tasks^50^. Conceptually, team cohesion plays a pivotal role in distinguishing effective teams from ineffective ones^51,52^. Moreover, there is evidence supporting the positive relationship between team cohesion and team performance^53,54^. To explore the effects of team cohesion on team performance in our setting, we could analyze the same players over multiple matches and observe whether poor results erode the cohesion between them, potentially leading to a player switching to another team.

### Team communication

Effective and open communication within teams is crucial for accomplishing targets and other team tasks^55^. Communication is frequently cited as a team process that correlates positively with performance and effective team functioning^51^. However, due to privacy requirements of the API, we cannot access the communications between players. However, this may be possible in other settings, for example in tournaments where players are recorded as they play. Analyzing this communication data can provide insights into the level of communication within each team and its potential impact on team performance, warranting further investigation in future research.

### Performance feedback

The model proposed by Ring and van de Ven^56^ suggests that cooperative relationships in teams evolve or dissolve over time based on performance feedback and evaluations of decisions made by team members. However, there is limited research on the influence of performance feedback mechanisms on cooperation^57^. Using our setting, researchers could assess whether good performance at the beginning of the match increases the likelihood of other team members following or imitating the successful performer.

### Performance landscapes and NK models

NK models for combinatorial complex tasks create performance landscapes based on N choice variables and K interactions in the payoff function^58–60^. Despite ontological challenges when applying natural science models in management^61^, Levinthal’s work^62,63^ has encouraged the adoption of NK modeling in management and organizational studies, spanning decision-making^64^, new product design^65^, organizational change^64^, and product modularity^66^. Numerous management papers have employed NK models, and these models provide a space of combinatorial alternatives where an alternative comprises N binary choice variables affecting its payoff value. The performance landscape created by the NK model represents the mapping of attributes to corresponding payoff values. Peaks in this landscape, denoted by K, correspond to local optima with specific payoffs. In our context, peaks with high payoff represent risky areas with abundant resources. By analyzing data from similar games, researchers can investigate how individuals or teams react, search, and navigate in different landscapes characterized by varying peaks and payoffs, shedding light on human decision-making and strategic behaviors^67^.

### Other study settings

In expanding upon our framework, future research can explore the interplay between behavioral interdependence and collaboration in a variety of organizational settings. For example, in the context of a multifaceted enterprise, researchers can consider the interaction between disparate departments, such as marketing and finance. The distinct nature of their functions may result in a low degree of behavioral interdependence; concurrently, their collaboration might remain minimal due to the segregated nature of their operations. Conversely, an alliance of independent firms uniting for a singular promotional initiative may each contribute unique resources while maintaining autonomy in their operations, epitomizing low interdependence. Yet, the collective endeavor necessitates a high degree of collaboration to ensure cohesive branding and communication. Moreover, in an academic institution, faculty within a department may share moderate interdependence due to shared governance responsibilities, while exhibiting lower collaboration, as their academic and research activities are conducted largely in isolation. Furthermore, a juxtaposition emerges in the case of project teams comprising diverse expertise. Here, a moderate level of interdependence is inherent as each member's output partially depends on others, coupled with a commensurate level of collaboration, which facilitates the integration of their specialized knowledge towards the project's objectives.

## Conclusion

In summary, our research offers significant contributions to the fields of behavioral science, team dynamics, and the study of e-sports. We replicate the curvilinear relationship between behavioral interdependence and team performance, demonstrating that the relationship is moderated by experience. Low-experience teams benefit from a loose, high behavioral interdependence strategy, while high-experience teams thrive in conditions of tight collaboration and high behavioral interdependency. This work addresses concerns related to the "replication crisis" and rising publishing costs in behavioral research by leveraging e-sports data, providing a cost-effective, granular, and highly accessible resource for examining complex human behaviors and team dynamics. It emphasizes the importance of embracing new research opportunities in the digital era and underscores the potential for bridging the gap between behavioral studies and dynamic, controlled e-sports environments to enrich our understanding of human behavior, team dynamics, and performance in digitally mediated contexts.

## Methods

We used the PUBG API to download various types of game data. More specifically, we collected two types of data: match telemetry data and player data. The first one, match telemetry contains events that happened inside the match. From this dataset we collected the following events:

*Location of players at different points in time*. Location is the main variable we use to assess collaboration within the team. This variable is defined as the location of every player every 10 s. This means that, for each player in the match, we have their location starting at second n (between 1 and 10) and, from then on, we have their location every 10 s (n + 10, n + 20...).

*Parachute landing location*. Landing location of each player is another important piece of information, as it serves as our measure of initial risk. As most players tend to land in locations where they can find initial weapons and other resources, we can infer that they consider these locations valuable and, therefore, these areas are risky. We used this data to create heatmaps that are used to assess riskiness.

*Match information* (ranking, type of match, map, players in the match). This data is needed for technical purposes, as we need to identify how player ranked throughout the match, which map they are playing on, and which players were in that match.

The second type of data we collect is player data. We use it to get information about the experience of the players to later analyze how it affects performance. A player’s experience is measured with rank points for each game season. Every season, each player in the game increases their rank points by getting good ranks, killing other players, and other actions that characterize the experience of the player. For that reason, we decided to take this piece of data as a control variable for the experience of the player. In total, we gathered data from 8866 squad-mode matches involving 140,699 teams composed by 562,796 players, which accounts for over 1.5 million hours of gameplay. The cumulative data volume amounts to approximately 130 GB.

### Team performance

In this paper, we assess team performance using the final ranking position (as well as achieving a top 5 or top 10 position for robustness checks, see Annex). Data on rankings was collected through a match telemetry API endpoint.

### Team collaboration and behavioral interdependence

Considering the game's nature and the data we collected, we defined both behavioral interdependence and team collaboration as the team dynamics regarding the physical distances between team members and their tendency to cluster together. This is consistent with previous behavioral research where physical proximity plays a key role in team members propensity to collaborate^68,69^. In general, existing evidence shows that team members who are physically close to each other are more prone to collaborate compared to those members with low physical proximity. Therefore, similarly to other papers on team dynamics^70^, we employed distance as a measure to analyze team collaboration and behavioral interdependence across teams. In particular, we defined two variables:

*Behavioral Interdependence: Distance to team centroid.* These are the distances of every player to the Euclidean centroid of the team at the point in time. In order to get this, we first found the centroid by getting the mean of the X and Y coordinates of each player at each point in time and then calculated the Euclidean distance between every player and that centroid. Then, we used DBScan algorithm with a minimum density of 1, so that a single player playing far from the rest could be considered a cluster, and 100 m epsilon. This threshold distance was inferred by looking at the distribution of the distances among the players for several matches, where we observed that the mean was around 100 m. Using the clustering results, we established three behavioral interdependence clusters for each situation: “Behavioral Interdependence High” when all players were within the same cluster, “Behavioral Interdependence Medium” when some players were in different clusters, and “Behavioral Interdependence Low” when each player represented their own isolated cluster. This approach allowed us to comprehensively categorize team behavioral interdependence levels.

*Team Collaboration: Pair distances.* These are the distances between any two players inside a same team at every point in time defined in spans of 10 seconds. Therefore, for a team of 4 people we had 6 data points at every point in time. We used a logarithmic form for the distance variable, following the logic that variation of distance on closer distances matters more than variation in further distances. Furthermore, we normalized distances because of the nature of the game. As we said previously, the playground gets smaller as the game goes on, restricting the movement of the players and incentivizing them to confront other teams. As this happens, intra-team distances get smaller as well, posing a risk of data leakage as it may wrongly signal that teams are closer (collaborating more). Hence, we normalized the distances by time remaining to avoid the confounding effect of the shrinking playground. These pair distances serve as our measures of “Collaboration Low” (this measure represents the farthest distance between any two players within the team, reflecting how spread out or dispersed the team is during the match, useful to identify more individualistic playstyle) and “Collaboration High” (this represents the closest distance between any two players within the team, indicating if they are moving together as a group).

The aforementioned operationalizations have several advantages in our research context. Our operationalization of behavioral interdependence captures the idea that individual players have the freedom to decide their behavior, being able to choose to play either close to each other or apart. This decision has important implications for players and their teams. For example, playing at a distance may enhance the chances of finding more resources through exploration but also increases the risk of encountering another team that is playing together, leading to a disadvantage of facing four players alone. Conversely, playing in close proximity raises the probability of surviving an attack from another team and enables micro-strategies, such as surrounding enemies.

Regarding our operationalization for team collaboration, virtual teams are often associated with communication difficulties due to lack of social and nonverbal cues^70^. In our setting, resources and enemies can be seen or heard at a given distance. For example, you can only hear shootings being fired at 150 m or less from the player location^21^. If team members are playing close to each other, they are being exposed to the same input information (i.e., hearing a shot or spotting an enemy), therefore facilitating coordinated actions; that is, team collaboration.

**Figure 3:**
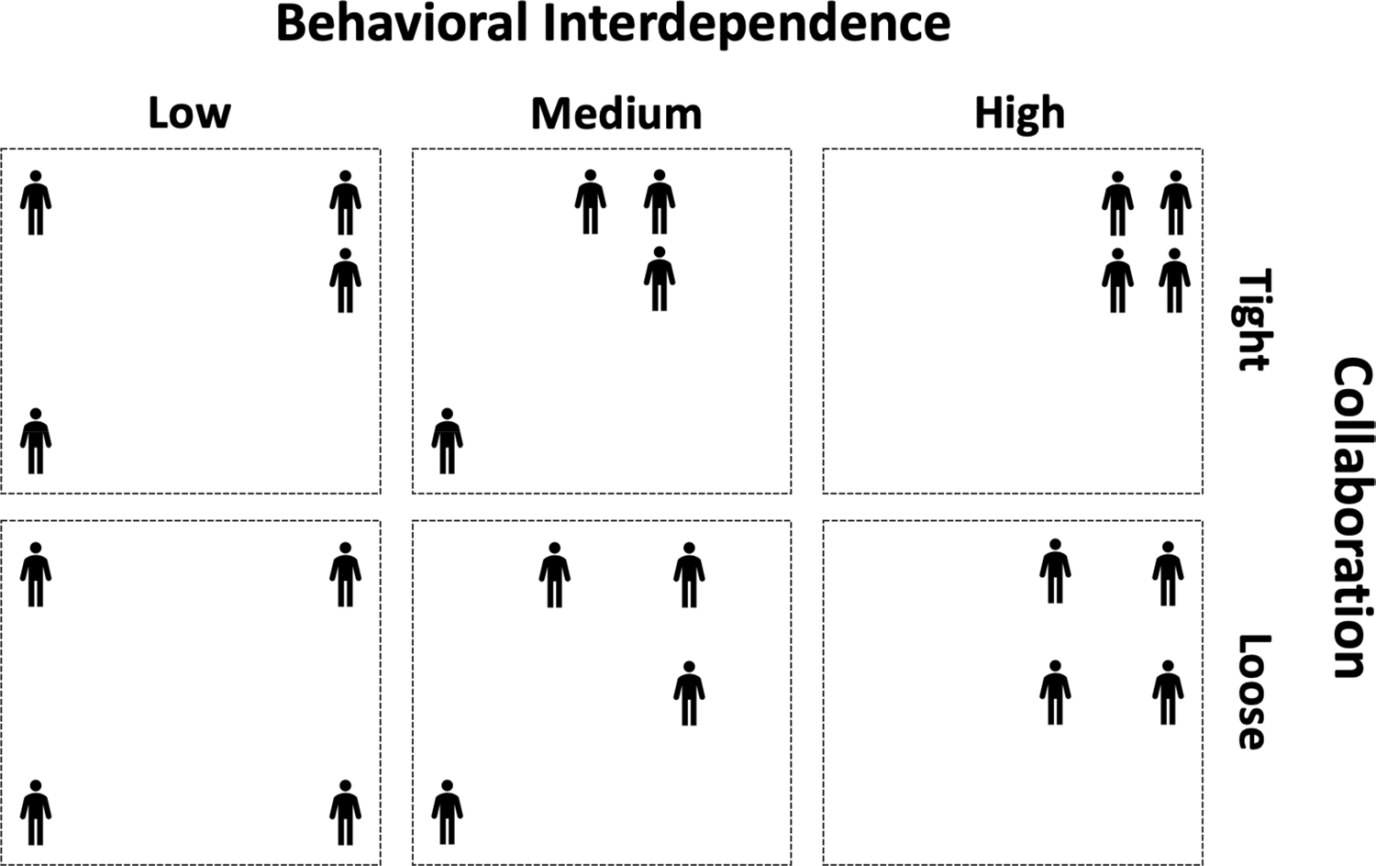
Schematic representation of team behavior at two intersecting levels: interdependence and collaboration.

Moreover, our operationalization allows us to analyze team behavior at two levels. First, at the behavioral interdependence level, and secondly at the collaboration level. For example, teams showing high behavioral interdependence can still have low collaboration within their cluster (see Fig. 3 for a schematic summary). This is what we call “loose behavioral interdependence” (when collaboration is low but behavioral interdependence is high). On the other hand, we call “tight behavioral interdependence” to team behaviors that are defined by high collaboration and high behavioral interdependence.

### Team risk taking

**Figure 4:**
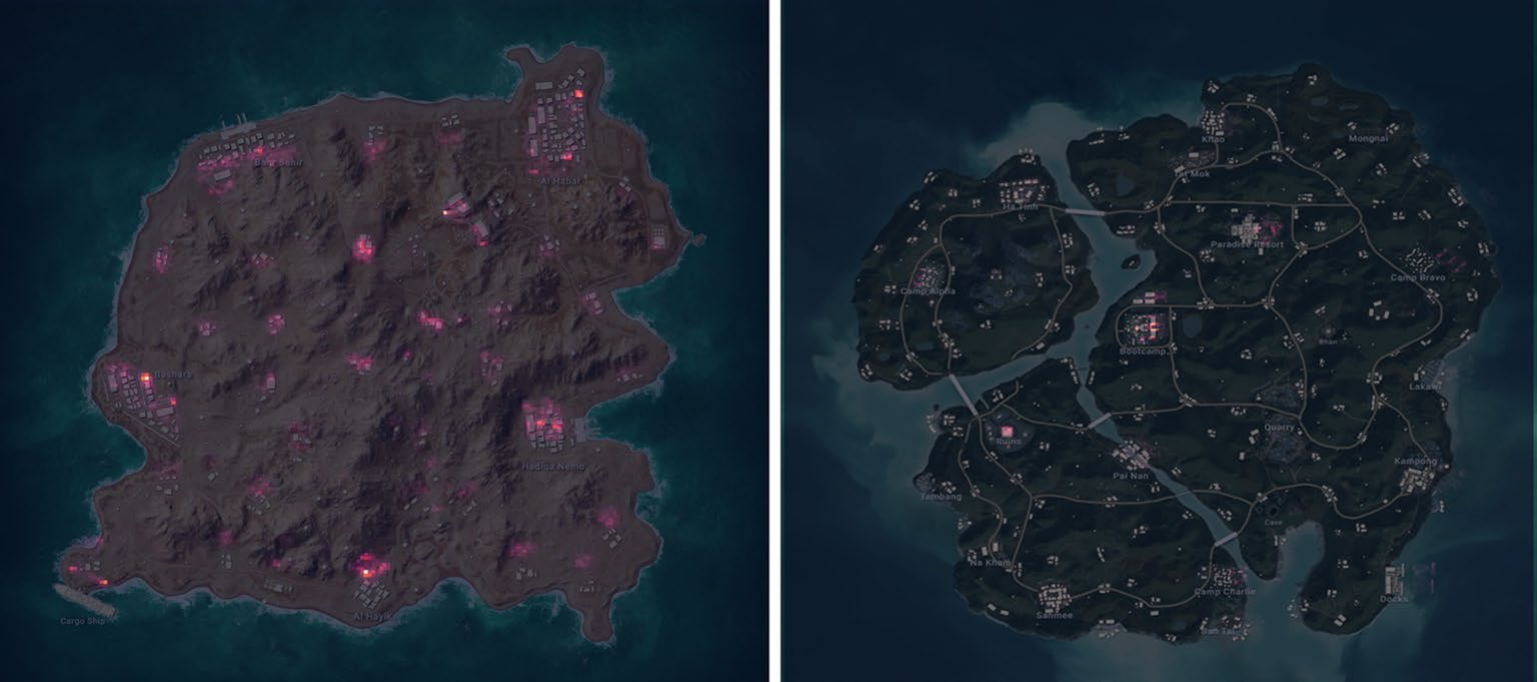
Landing hotspots. Karakin (left) and Sanhok (right).

In PUBG, resources are distributed unevenly across the island, with certain locations, such as factories and cities, containing more resources compared to isolated areas like farms or small villages. However, the potential for higher rewards in these resource-rich locations also comes with increased risks. To measure the level of riskiness in different areas, we determined the optimal size of a chunk as 10 by 10 m. To achieve this, we created a grid of 10 by 10-m cells for each map, resulting in 816 by 816 cells for Miramar and Erangel, 408 by 408 cells for Sanhok, and 204 by 204 cells for Karakin. Subsequently, we assigned to each cell the number of players landing in that specific area, which served as our operationalization of risk (see Fig. 4 for an example of the risky areas). We obtained this data from a sample of 50,000 players and their corresponding landings. By employing this approach, we were able to quantitatively assess and analyze the diverse risk levels across different regions of the maps, forming the foundation for our risk variables.

*Initial risk taking—Landing Risk.* In our empirical setting, we capitalize on the fact that all players start the match in a plane, giving them the freedom to choose their landing location using a parachute. This unique opportunity enables us to analyze their risk-taking behaviors in the context of initial input gathering. While risk-averse teams often opt for isolated areas, many others decide to take the gamble of landing in resource-rich regions. This decision creates a pivotal trade-off: should the team risk landing in a resource-rich area with the possibility of encountering numerous other teams vying for the same resources, or should they play it safe and choose low-resource areas? This trade-off serves as the basis for operationalizing risk-taking (or risk aversion) through the decision made when selecting the landing spot. In our study, we quantified two variables related to risk-taking in team behavior: “High risk landing” and “Low risk landing.” The “High risk landing” variable captures the highest level of landing risk that each team took, reflecting their willingness to land in resource-rich areas despite the potential challenge of encountering other teams. On the other hand, the “Low risk landing” variable represents the lowest landing risk observed for each team, signifying their choice to play it safe by landing in low-risk areas with fewer resources and potentially fewer opponents. These variables provide valuable insights into the risk preferences of teams and their strategic decision-making during the initial stages of the match.

*Overtime risk taking*. To emphasize the importance of longitudinal efforts and account for temporal aspects in teams^19,71^, we incorporated risk heatmaps as a measure of risk-taking at each point in time during the match. This approach allowed us to calculate the risk that team members were taking at each moment in their pursuit of gathering inputs for the team. By analyzing this data, we could determine whether teams consistently avoided risky areas throughout the entire match, moved from one risky area to another to maximize input gathering, or employed a mixture of both strategies. This valuable information was captured in our "High risk" and "Low risk" variables, where we calculated the time spent in high-risk areas and low-risk areas for the most and least risk-taking members of the team, respectively. Using these measures we quantify how much time teams dedicate to risk-taking actions. If a team spends a significant amount of time in high-risk areas, it indicates a higher propensity for risk-taking behavior. On the other hand, if they primarily remain in low-risk areas, it suggests a more cautious and risk-averse strategy. By analyzing these time-based metrics, we gain insights into the persistence and consistency of risk-taking behavior over the course of the match.

*Control variables.* In order to accurately assess the effects of collaboration and risk-taking on team performance, we implemented several control variables. To account for variations in experience among teams, we included the rank points of each player for the season as a control variable. These rank points represent the players' skill levels and provide valuable insights into the expertise of the team members. To control for collaboration dynamics, we incorporated match-level control variables, such as the mean of maximum and minimum distances across all teams in each match. These variables offer an overview of overall collaboration patterns within the game and help us understand variations in collaboration across different matches. Additionally, we utilized the average standard deviation of distances across teams within each match, providing a more nuanced understanding of how collaboration is dispersed among teams in the same match.

**Figure 5:**
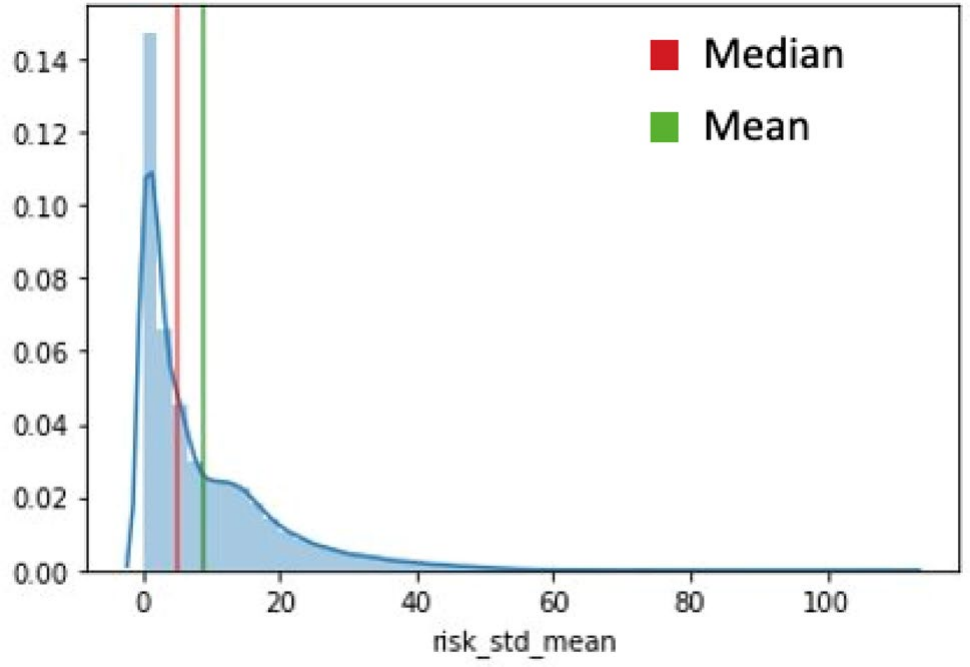
Risk distribution.

Regarding risk variables, we introduced the average risk level of the match across all teams as a control variable. This measure allowed us to consider the overall risk context in the game and assess team risk-taking behaviors in relation to the general risk environment. Furthermore, we examined the average strategies employed by each team in the match, focusing on specific risk-related strategies like exploration, ambidexterity, and exploitation. These risk strategies shed light on how different approaches to risk might impact team performance outcomes. We also calculate the risk category. Team processes in the game can exhibit varying orientations, focusing on either input (resource) gathering or output (performance) at different points in time. To determine each team's risk category, we analyzed the distribution of the risk variable described before (see Fig. 5). To identify an appropriate segmentation point, we considered both the median and mean values. Ultimately, we chose the median as it more intuitively captured a significant number of teams, while the mean encompassed over 75% of the teams, which was not a meaningful segregation for our analysis.

**Figure 6:**
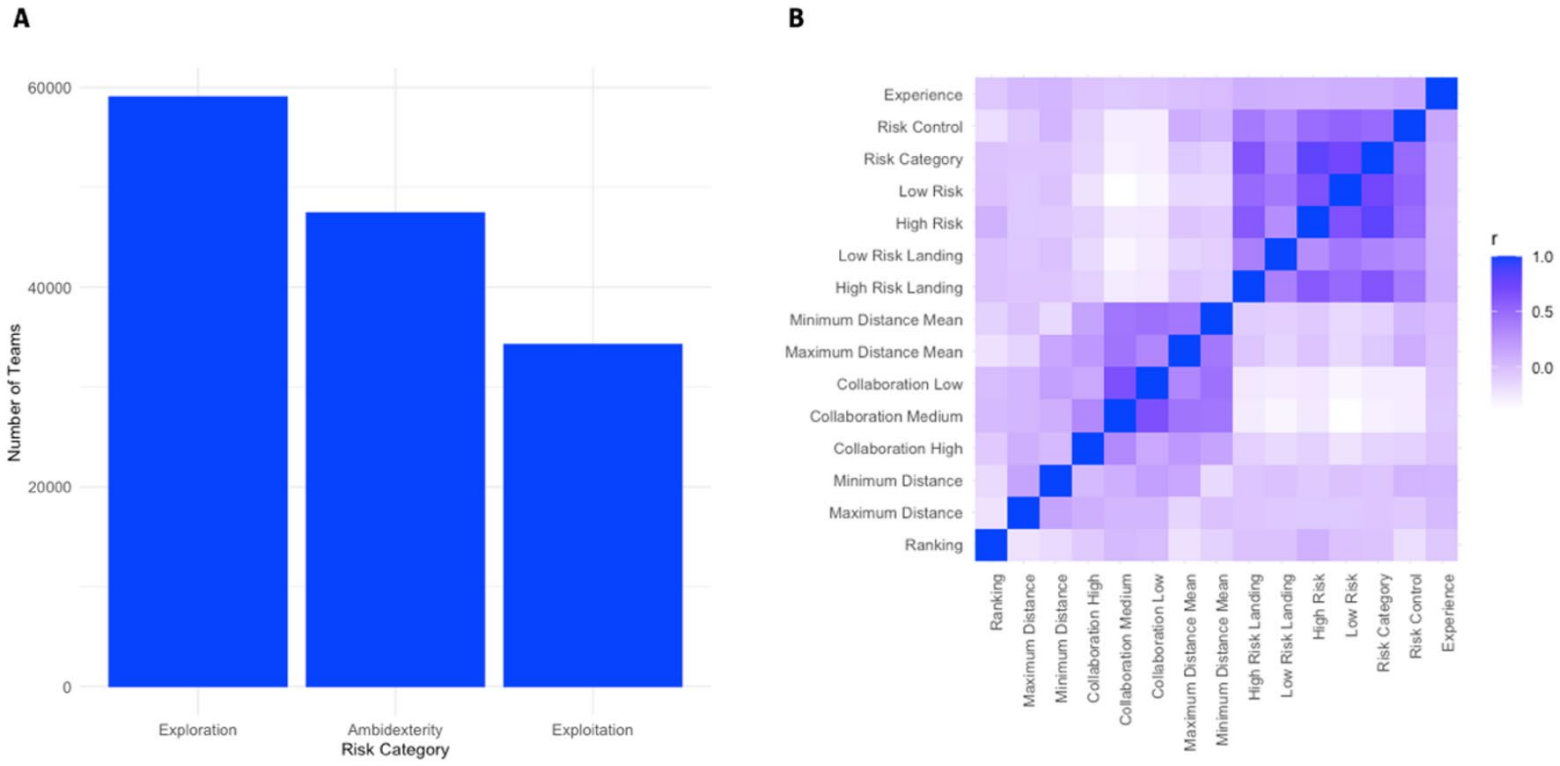
(**A**) Count of teams within each risk category. (**B**) Correlation matrix of all variables included in the models.

Therefore, we used the median as the threshold to distinguish the low-risk strategy (risk category = -1), the medium risk strategy (risk category = 0), and the high-risk strategy (risk category = 1) comprised all the remaining teams with high values in their risk distribution. See Fig. 6A for the distribution of risk strategies and Fig. 6B for the correlations among all variables in the models.

By incorporating these control variables, we aimed to minimize potential confounding effects and ensure that our findings on collaboration and risk-taking remain robust and accurate, providing a comprehensive and reliable analysis of team performance dynamics in the context of the game.

*Outliers and robustness checks.* To address the potential influence of outliers, particularly those likely associated with hackers or game bugs, we employed the Z-score method. By setting a threshold of 3 standard deviations, any value that deviated significantly from the mean of the variable was identified as an outlier and subsequently dropped from the dataset. This approach allowed us to mitigate the impact of irregular data points and ensure the integrity of our analysis. Following this outlier removal process, we arrived at our final sample size of 140,699 players. The prevalence of outliers in our data can be attributed to two main factors. Firstly, the presence of hackers in the game, who exploit bugs or cheats to gain an unfair advantage, leading to anomalous data points. Secondly, the inclusion of game bots by PUBG in each match, which behave randomly and do not adhere to any specific strategy, contributing to random scattering of data points throughout the maps.

One potential confounding variable in our study could be the prior conjoint experience of players, wherein a team is comprised of players who have previously played together, suggesting potentially enhanced coordination among them. To investigate this, we defined conjoint experience as the frequency with which a player ID has played in the same team as all other player IDs. We assessed this by calculating the proportions of dyads (two-player conjoint experience), triads (three-player conjoint experience), and quartets (four-player conjoint experience). Our analysis revealed that only 0.57% of players in our sample have conjoint experience with other players in the same dataset. Specifically, 0.52% of players have dyadic conjoint experience, 0.03% have triadic conjoint experience, and 0.02% have quartet conjoint experience. These findings instill confidence that the influence of this potential confounding factor is expected to be minimal, thus reinforcing the robustness of our study’s outcomes.

### Model estimation

In our analysis, we employed ordinal models. In there, we utilized the ranking position at the end of the match as our dependent variable, conducting an ordinal linear regression. Following previous team research^15^, we use the approach suggested by Aiken and West^72^ to estimate simple slopes moderated by experience level. We do so through a stratified analysis with three clusters: low experience, medium experience, and high experience. In order to classify the players in each of the three groups, we employ the K-means algorithm. This method identified 85,388 players with low experience, 30,645 players with medium experience, and 24,666 players with high experience. The mean experience (and range) for each group is: $${\upmu }_{low experience} =$$ 0.013 [0.000–1.685], $${\upmu }_{medium experience} =$$ 3.358 [1.686–4.136], $${\upmu }_{high experience}$$$$=$$ 4.914 [4.136–75.599].

Using the ranking positions and variables of each three experience levels, we calculated five distinct ordinal models. The first model incorporates only low experienced players, the second one only medium experienced players, and the third one high experienced players. The fourth and fifth models are robustness checks for the statistically significance of the variables of interest (behavioral interdependence and experience). The fourth model includes the interaction effect between behavioral interdependence and experience, while the fifth model includes quadratic terms for the behavioral interdependence variables (to confirm a curvilinear effect). Furthermore, in order to maintain the parsimony of the tables in the main document, the results of the fourth and fifth models, and the detailed results of control variables are presented in the Annex.

For the variance explained in each model, we employ Maximum Likelihood R-squared (r2ML), which is an extension of the traditional R2 used in linear regression to the ordinal setting. It quantifies the proportion of variance in the dependent variable that is accounted for by the model's predictors. It ranges from 0 to 1, where 0 indicates that the model does not explain any variance, and 1 indicates a perfect fit where the model completely explains the variance in the data. Therefore, r2ML provides an assessment of the model's goodness of fit and how well it captures the underlying patterns in the data.

To ensure the validity and reliability of our models, we carefully examined the variance inflation factors (VIF) of all variables. We found that all VIF values were below 5, which indicated the absence of multicollinearity among the predictor variables. This provided confidence in the robustness of our models and the accuracy of the estimates obtained, allowing us to draw meaningful conclusions regarding the impact of various factors on team performance in PUBG.

### Supplementary Information

Supplementary Information.

## Data Availability

The datasets generated during and/or analysed during the current study are available in the Harvard Dataverse repository, https://doi.org/10.7910/DVN/W2JPT3. All the raw data and code to clean, process, analyze, and visualize the data is available in the following GitHub repository: https://github.com/ccfarre/PUBG/.
