# Supplementary material for: Experience shapes non-linearities between team behavioral interdependence, team collaboration, and performance in massively multiplayer online games
Source: Sci Rep. 2024 Apr 3;14:7850. doi: 10.1038/s41598-024-57919-w (PMC10991398; doi:10.1038/s41598-024-57919-w)
Supplement: Supplementary file 1 — Supplementary Information. [file 41598_2024_57919_MOESM1_ESM.docx]

Experience Shapes Non-Linearities Between Team Behavioral Interdependence and Performance in Massively Multiplayer Online Games

- ANNEX -

**Methods for Logistic Models**

On the other hand, for the logit models, we created dummy variables to represent whether the team achieved a top 10 or top 5 position in the match. These two dependent variables were characterized by a Bernoulli distribution, and we applied a logistic regression model for the analysis. We constructed a total of six logistic models to examine the relationships between the variables and the two dependent variables: top 10 and top 5. The first logistic model encompassed all the variables, exploring their effects on achieving a top 10 ranking in the match. Similarly, the second model included all variables but focused on the top 5 dependent variable. The third and fourth logistic models specifically analyzed the collaboration and risk variables, respectively, in relation to the top 10 ranking, while accounting for the relevant control variables. Models five and six were replicas of models three and four, respectively, but targeted the top 5 dependent variable.

For the variance explained in each model, we employ Tjur's R2. Tjur's R2 is a measure of the proportion of explained variation in a binary logistic regression model. Unlike Nagelkerke's R2, which applies to ordinal logistic regression, Tjur's R2 is specifically designed for binary (dichotomous) outcome variables. Tjur's R2 is based on the differences in predicted probabilities between the model and a reference model that includes only the intercept. It ranges from 0 to 1, with 0 indicating that the model does not explain any variation beyond chance, and 1 indicating a perfect fit where the model perfectly predicts the outcomes.

To ensure the validity and reliability of our models, we carefully examined the variance inflation factors (VIF) of all variables. We found that all VIF values were below 5, which indicated the absence of multicollinearity among the predictor variables. This provided confidence in the robustness of our regression models and the accuracy of the estimates obtained, allowing us to draw meaningful conclusions regarding the impact of various factors on team performance in PUBG.

**Logistic Models Performance and Results**


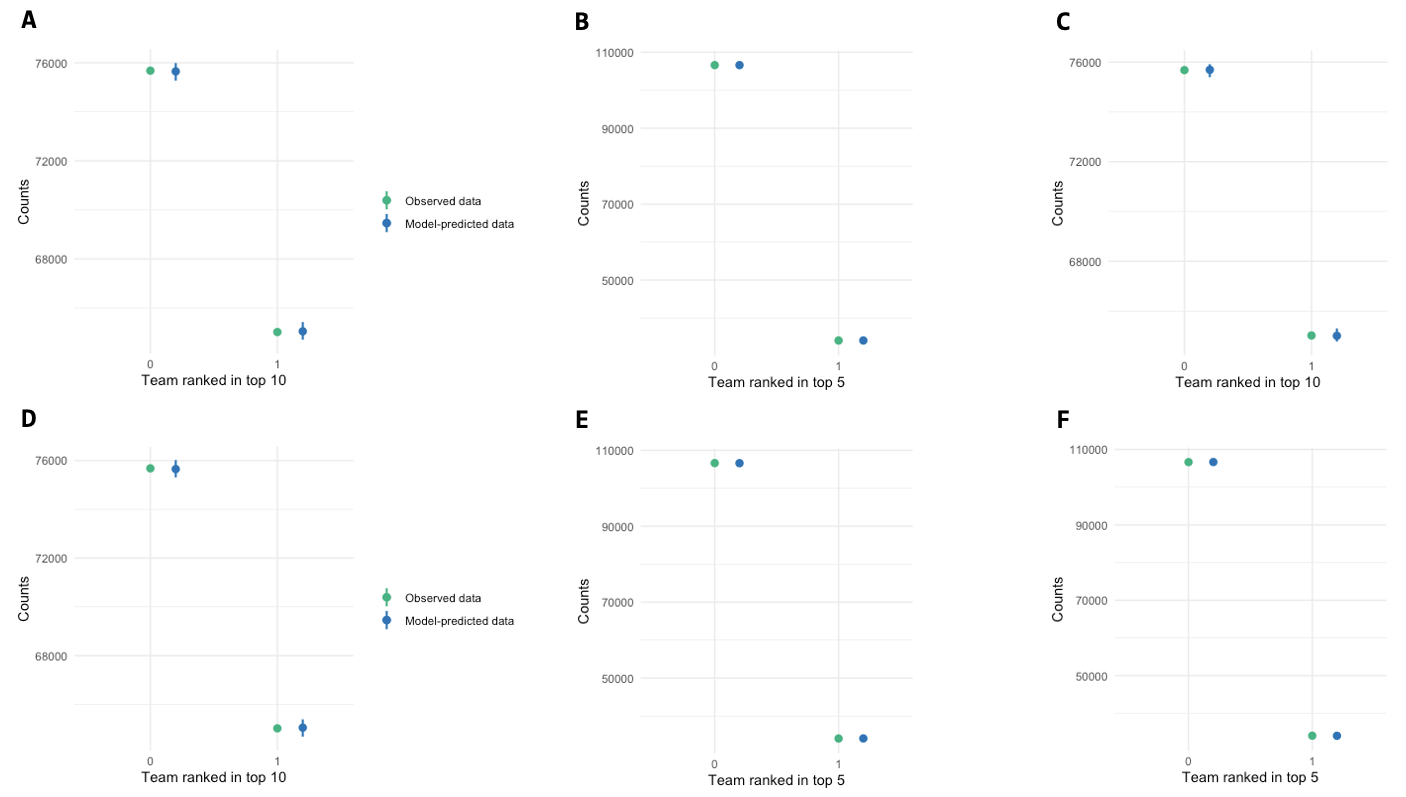


*Figure A1: Predicted VS Observed data in logistic models; (a) model 1 (all variables, top 10), (b) model 2 (all variables, top 5), (c) model 3 (logistic for collaboration variables and top 10), (d) model 4 (logistic for collaboration variables and top 5); (e) model 5 (risk variables and top 10), (f) model 6 (risk variables and top 5).*

Both logistic models 2 and 5 (all variables and top 5 as dependent variable, and risk variables and top 10 as dependent variable respectively) are the best models as they have lower AIC (1.3e+05, >.999; 1.4e+05, <.001), compared to the worst ones, logistic models 3 and 6 (1.6e+05, <.001; 1.4e+05, <.001), indicating better model fit and parsimony. Model 2 has the highest Tjur's R2 (0.146) among all models, showing better ability to explain variance in the data compared to the other models. Regarding predictive accuracy, logistic model 2 has the lowest RMSE (0.397), followed by logistic model 5 (0.405), outperforming logistic model 3 (0.437) and logistic model 6 (0.411). Furthermore, logistic model 2 has the highest PCP (0.687), indicating a higher percentage of correctly predicted outcomes, followed by logistic model 5 (0.675). Overall, logistic model 2 and logistic model 5 consistently show better performance across multiple metrics, including model fit, explanatory power, predictive accuracy, and correctly predicted outcomes, making them the preferred models for this analysis.


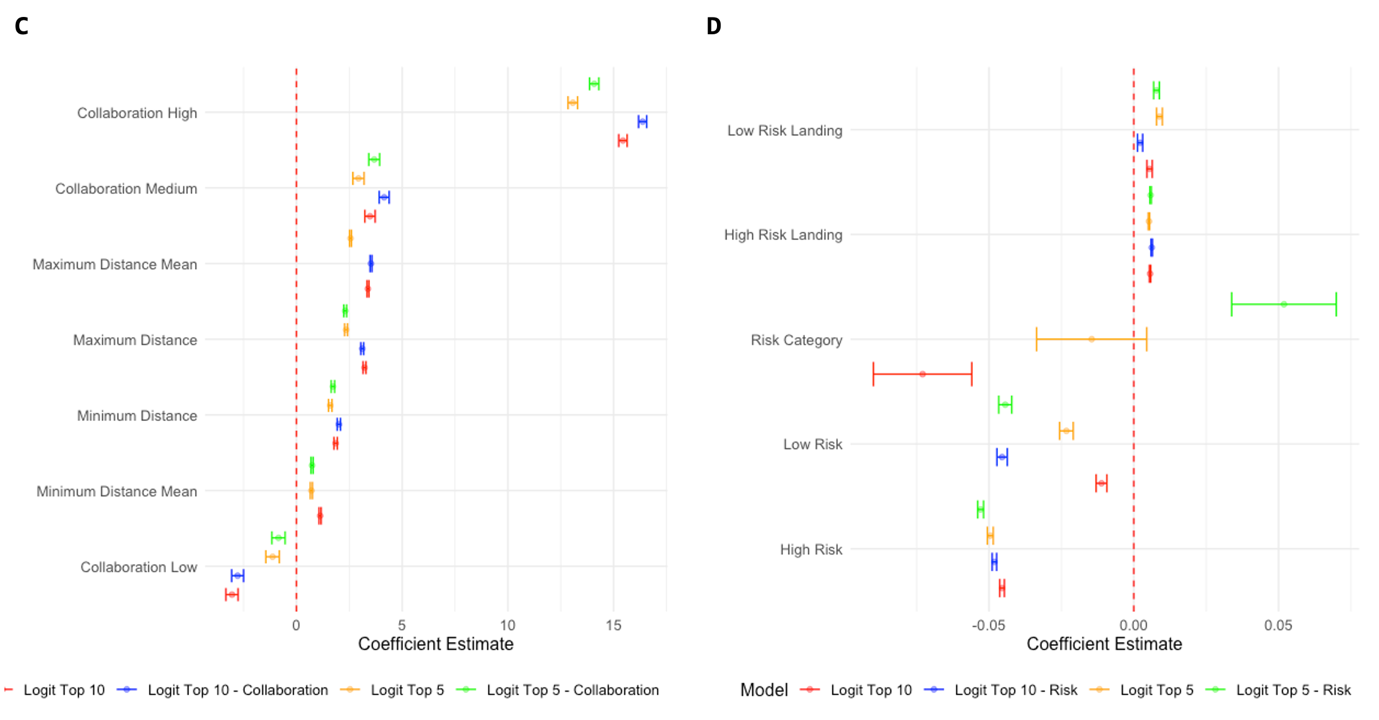


*Figure A2C: collaboration variables in logistic model 1 (all variables, top 10), 2 (all variables, top 5), 3 (logistic for collaboration variables and top 10), and 4 (logistic for collaboration variables and top 5); Figure A2D: risk variables for logistic model 1 (all variables, top 10), 2 (all variables, top 5), model 5 (risk variables and top 10), and 6 (risk variables and top 5).*

The results should be interpreted as higher coefficient estimates being associated with higher probability of ending the match in the Top 5 or Top 10 teams. Like in the ordinal models (Figures A2C and A2D), teams that collaborate closely tend to have a much higher chance of ending up in the top 10 positions (*β =* 15.44, p-value < 0.001). Complementarily, teams with low collaboration are likely to perform poorly and are less likely to be in the top 10 (*β =* -2.82, p-value < 0.001). Adding robustness to these results, medium collaboration is also associated with positive performance, although to a lesser extent than high collaboration (*β =* 3.51, p-value < 0.001). The results in the logistic model for teams ending up in any of the Top 5 positions yields similar results. Teams with higher collaboration are more likely to be in the top 5 positions (*β =* 13.06, p-value < 0.001). On the other hand, teams with lower collaboration have a negative impact on their chances of being in the top 5, but the effect is smaller than in the top 10 model (*β =* -0.87, p-value = 0.006). As for teams with moderate collaboration, they also have a positive impact on their chances of ending up in the top 5 (*β =* 2.95, p-value < 0.001).

For the variable "Maximum Distance Mean," we observe significantly positive coefficient estimates in both models (*β =* 3.40 for top 10 and *β =* 2.57 for top5). For the variable "Minimum Distance Mean," we also observe significantly positive coefficient estimates in both models, "Logit Top 10" (*β =* 1.12) and "Logit Top 5" (*β =* 0.71). For the variables "Maximum Distance" and "Minimum Distance," we again observe positive coefficient estimates in both models (*β =* 3.23 for maximum distance and *β =* 1.87 for minimum distance in the top 10 model, and *β =* 2.36 and *β =* 1.62 for the top 5 model), indicating that an increase in the maximum and minimum distances between players is associated with a higher likelihood of better rankings. Overall, like in the ordinal models, the results of the logistic models consistently show that increasing the distances between players, whether considering the mean distances or the maximum and minimum distances, is positively associated with better rankings. Moreover, the effect is generally stronger in the "Logit Top 10" model, indicating that these distance-related variables play a more substantial role in predicting top 10 rankings compared to top 5 rankings.

Like in the ordinal models, the results suggest that teams aiming to optimize their performance should emphasize close collaboration among players, but with a critical consideration of a threshold point. Beyond this point, the benefits of maintaining very close collaboration may diminish. Instead, teams may achieve better outcomes by maintaining collaboration within clusters (all players within the same cluster) but allowing for higher distances between these clusters. This balance between proximity and distance seems to have a more significant positive impact on team performance, leading to higher rankings in the final standings.

As for risk, in the top 5 model, teams taking higher landing risks are significantly more likely to be in the top 5 positions (*β =* 0.006, p-value < 2.2e-16); similarly, as in the other models, teams taking lower landing risks are significantly more likely to be in the top 5 positions (*β =* 0.009, p-value < 0.002). Moreover, our results point out towards the idea that teams facing higher overall risks are significantly more likely to end up in lower rankings (*β =* -0.046, p-value < 0.002). Similarly, teams facing lower overall risks are significantly more likely to have lower rankings (*β =* -0.015, p-value < 0.000).

The estimate for "Risk Category" shows that teams applying ambidexterity strategies (finding an equilibrium between exploration and exploitation strategies) tend to have significantly higher rankings positions compared to teams purely adopting other strategies like exploitation or exploration alone as shown by the estimate close to 0. This is the case both in the top 10 and to 5 models (*β =* 0.131, p-value < 0.000 and *β =* 0.195, p-value < 0.000 respectively) means that teams following exploitation strategies have an estimated log-odds increase of approximately 0.131 for being ranked in the top 10, compared to teams following other strategies.
